# Territorial-sneaker games with non-uniform interactions and female mate choice

**DOI:** 10.1093/beheco/araf002

**Published:** 2025-01-17

**Authors:** Thomas N Sherratt, Christopher D Beatty, Ian Dewan, Katherine Di Iorio, Isaac Finkelstein, Karl Loeffler-Henry, Marrissa Miller, Falisha Para, Megan Raposo, Frances Sherratt

**Affiliations:** Department of Biology, Carleton University, 1125 Colonel By Drive, Ottawa, ON, Canada, K1S 5B6; Program for Conservation Genomics, Department of Biology. Stanford University, Jane Stanford Way, Stanford, CA 94305, United States; Department of Theoretical Biology, Max Planck Institute for Evolutionary Biology, August-Thienemann-Str. 2, 24306 Plön, Germany; Department of Biology, Carleton University, 1125 Colonel By Drive, Ottawa, ON, Canada, K1S 5B6; Department of Biology, Carleton University, 1125 Colonel By Drive, Ottawa, ON, Canada, K1S 5B6; Department of Biology, Carleton University, 1125 Colonel By Drive, Ottawa, ON, Canada, K1S 5B6; Department of Biology, Carleton University, 1125 Colonel By Drive, Ottawa, ON, Canada, K1S 5B6; Department of Biology, Carleton University, 1125 Colonel By Drive, Ottawa, ON, Canada, K1S 5B6; Department of Biology, Carleton University, 1125 Colonel By Drive, Ottawa, ON, Canada, K1S 5B6; Department of Neuroscience, Carleton University, 1125 Colonel By Drive, Ottawa, ON, Canada, K1S 5B6

**Keywords:** alternative reproductive tactic, asymmetric game, character displacement, *Mnais* damselfly, polymorphism, replicator dynamics, sneaker, territorial

## Abstract

Male territorial-sneaker polymorphisms are common in nature. To understand how these polymorphisms evolve, we developed a game theoretical model analogous to the classical Hawk-Dove model, but with two important differences. First, we allowed non-uniform interaction rates of strategies to account for the possibility that some interactions between male strategies are disproportionately more frequent than others. Second, we allowed females to exhibit a preference for one type of male and thereby choose mates adaptively. Selection dynamics were modeled using coupled replicator equations. The model confirms that there is a broad range of conditions under which a male polymorphism will arise. We applied the model to understand the genetic polymorphism in adult male *Mnais* damselflies (Zygoptera). Here, orange-winged adult males defend oviposition sites and mate with females when they arrive, while clear-winged ‘sneaker’ males are typically non-territorial and opportunistically mate with females. Intriguingly, in allopatry, the males of *Mnais costalis* and *M. pruinosa* both exhibit the same orange-clear winged polymorphism but where the species co-occur, males of *M. costalis* evolve orange wings while males of *M. pruinosa* tend to evolve clear wings. To understand this phenomenon and evaluate the importance of female choice in mediating it, we extended our game-theoretical model to two interacting species. While both competitive and reproductive interference can explain the male monomorphisms in sympatry, reproductive interference explains the phenomenon under a wider set of conditions. When females of the rarer species change their male preferences to facilitate species discrimination, it can generate runaway selection on male phenotypes.

## Teaser

Many species exhibit specialized territorial males and opportunistic sneakers. Here we present a game-theoretical model to help understand how this polymorphism arises. We make two key assumptions. First, we allow certain male strategies to interact disproportionately. Second, we allow female choice. The model readily explains the orange- and clear-winged polymorphism found in *Mnais* damselflies. It also suggests that males of co-occurring *Mnais* species evolve distinct morphs due to females choosing mates based on species identity.

## Introduction

There are many examples of species with more than one discrete form of male, with each male type exhibiting an alternative reproductive tactic (“ART,” Gross 1996; Shuster and Wade 2003; Taborsky et al. 2008; Neff and Svensson 2013; Schradin 2019). For example, “independent” male ruff (*Philomachus pugnax*) court females by defending mating territories, while co-occurring “satellite” male ruff tend to occupy areas on the boundaries of territories and attempt to sneak copulations when the territory holder is distracted (Widemo 1998). Likewise, the males of several salmonid species occur in two forms—large dominant “hooknoses” that defend breeding grounds and court females, and small subordinate “jacks” that lurk near these breeding grounds and seek to fertilize the female’s eggs after it spawns (Gross 1996).

In many species in which the above territorial-sneaker tactics have been reported, the male form that develops is determined solely by the male’s environment or by a gene-environment interaction (Plaistow et al. 2004; Neff and Svensson 2013). For example, the quantity and quality of food that larvae receive during development affects whether males of the dung beetle (*Onthophagus taurus*) develop horns (used in combat) or not (Moczek 1998). In the above instances, conditional selection (eg if you are small, fighting horns are not favored) can readily explain the maintenance of variation in the male phenotype. However, in other instances the male form is genetically determined. For example, males of the marine isopod *Paracerceis sculpta* occur in three different heritable forms, each with different reproductive tactics, including one form that defends harems (Shuster and Wade 1991). In these instances, the co-existence of the different morphs is likely maintained by a form of negative frequency-dependent selection in which a morph is favored when relatively rare but disfavored when relatively common.

To understand how territorial-sneaker polymorphisms can evolve and be maintained in a species under frequency-dependent selection, we need the tools of evolutionary game theory (Maynard Smith 1982; McNamara and Leimar 2020; Broom and Rychtář 2022). It has long been appreciated that territorial-sneaker interactions closely parallel the classical Hawk-Dove model in which Hawks fight aggressively for resources, and Doves back down (Maynard Smith and Price 1973; Maynard Smith 1982). Indeed, Tainaka et al. (2007) and Tanaka et al. (2009) both invoked this model when they attempted to explain variation in male reproductive tactics in the orangutan (mature adult vs. arrested adult) and salmon (hooknose vs. jack) respectively. Let T and S represent the territorial and sneaker strategy respectively and *W*(X,Y) represent the payoff to strategy X when played against strategy Y. It is well known that in any two-strategy game if *W*(T,T) < *W*(S,T) and *W*(T,S) > *W*(S,S) then a mixed set involving pure strategies (in which some males are territorial and other males are sneakers) is evolutionarily stable and will be converged on through replicator dynamics (Nowak 2006). While W(X,Y) > W(Y,Y) > W(Y,X) > W(X,X) with X = T, Y = S and *vice versa* represent the defining inequalities of the Hawk-Dove game (aka “Chicken” or “Snowdrift”), note that there are other inequalities (such as W(X,Y) > W(Y,X) > Y(X,X) > W(Y,Y) seen in the “Leader game,” Merrick and Shafi 2013) that would also render a mixed strategy set at equilibrium.

Classical two-strategy games can be informative, but there are at least two important implicit assumptions in the standard approach that limit its application to understanding the maintenance of male ARTs. The first limitation is that males with the two strategies are assumed to meet at random, with the relative probabilities of their interaction dictated simply by their relative densities. However, in many territorial-sneaker systems, territorial-sneaker interactions over a female may be much more common than territorial-territorial or sneaker-sneaker interactions, due to the spatial segregation of territory holders and the parasitic nature of sneakers. In an elegant paper, Taylor and Nowak (2006) extended the classical two-strategy model with uniformly distributed encounters to allow interaction rates to depend on strategies. Here, we show how their approach can be used to understand the polymorphisms that evolve in games involving ARTs. Second, and perhaps more importantly, many of the earlier games used to understand ARTs (Tainaka et al. 2007; Tanaka et al. 2009) have not explicitly considered pre- or post-copulatory female mate choice. However, females are not simply bystanders in the contest between territorials and sneakers and may exhibit their own strategic preferences. For example, in species with male ARTs involving external fertilization (such as salmonids), females appear to prefer mating with sneaker males (Watters 2005; Reichard et al. 2007). Arguably, a fixed female preference could be considered an implicit component of any parameter that partitions the fertilization success between territorial and sneaker males, but if female choice is dependent on the frequency of male morphs (see below) then it needs to be considered explicitly.

### Case example: *Mnais* damselflies

**Fig. 1.**
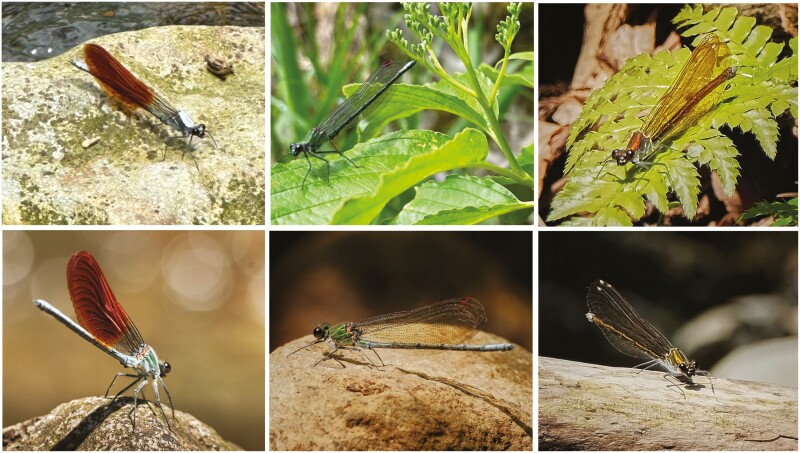
Top row, left to right: the orange-winged male, clear winged-male and female of *Mnais costalis.* Bottom row, left to right: the orange-winged male, clear winged-male and female of *Mnais pruinosa.* When allopatric, both species exhibit the same sex-limited color polymorphism with two types of male. In locations where the two species co-occur, then *M. costalis* males are predominantly orange-winged and *M. pruinosa* are predominantly clear-winged. Thus, there is a strong association between male polymorphism/monomorphism and whether the species are allopatric/sympatric (Tsubaki and Okuyama 2016). Photographs by IF and TNS.

A particularly intriguing example of sneaker-territorial mating tactics where both of the above assumptions are questionable is seen in damselflies of the genus *Mnais* (Zygoptera: Calopterygidae). The adult males of these damselflies are either orange-winged or clear-winged and crossing experiments have generated phenotypic ratios consistent with the morph type being genetically controlled at a single-locus (Tsubaki 2003; see Fig. 1). The orange-winged morphs tend to be territorial and defend suitable oviposition substrates, chasing away intruding males. By contrast, the clear-winged males are usually non-territorial and perch in concealed locations around occupied territories, attempting to copulate with any female they can intercept (Nomakuchi et al. 1984). Territorial disputes can be quite aggressive between orange-winged residents and adjacent territory holders, as well as between orange-winged residents and orange-winged intruders that have not yet secured a territory. Sneakers often fight to gain access to a suitable vantage point or to gain access to a female and these interactions also tend to be relatively aggressive (Nomakuchi et al. 1984). However, non-territorial clear-winged morphs tend to retreat quickly when challenged by territorial orange-winged morphs (Nomakuchi et al. 1984; Hooper et al. 2006). When a female arrives at a territory, the orange-winged resident sometimes courts her before seeking to form a tandem and copulate (Nomakuchi et al. 1984) then guards the female without physical contact while she oviposits (Siva-Jothy and Tsubaki 1989). By contrast, clear-winged males do not display to females or engage in post-copulatory mate guarding, possibly because it would attract the attention of the territorial male (Siva-Jothy and Tsubaki 1989).

The frequency of orange-winged morphs of *Mnais* damselflies is approximately 40 to 70% depending on time and location (Tsubaki et al. 1997; Tsubaki and Okuyama 2016), which is too high to be explained by recurrent mutation (Ford 1945). Since the estimated lifetime reproductive success of the two morphs is similar (Tsubaki et al. 1997) it is unlikely that the polymorphism is maintained by heterozygote advantage. Yet given how closely associated the strategies are with reproductive behavior, they are also unlikely to be selectively neutral. Nomakuchi and Higashi (1985) proposed a graphical model in which the reproductive success of both orange-winged males and clear winged-males on territories decline as favorable territories are successively filled. Since orange-winged-males were proposed to have consistently higher reproductive success than clear-winged males on any given territory, then the net effect would be selection for orange-winged males, whatever proportion of clear-winged males exceed a given “threshold of reproductive success.” However, the authors go on to advocate treating the evolved polymorphism as an evolutionarily stable strategy (ESS), and this is precisely the approach we have adopted here.

Non-uniform interaction rates between male morphs have already been observed in this system. For example, in a study of *Mnais pruinosa*, Nomakuchi et al. (1984) noted that “*the unilateral pursuit of strigata* [clear-winged] *males by esakii* [orange-winged] *was observed in overwhelming frequency*.” Indeed, the observed (non-experimental) frequencies of orange-winged-orange-winged (82 whether holding a territory or not), orange-clear (129) and clear-clear (25) interactions differ significantly from a binomial (X^2^ = 6.11, df = 2, *P* = 0.047). Female choice is also expected in *Mnais* damselflies, given the active role that female damselflies tend to play in mating—from deciding when and where to seek a mate, to choosing whether to accept the male’s sperm (Fincke 1997). Indeed, since *Mnais* females are frequently courted by orange-winged territory holders, it implies that females of this species can decline potential mates (either pre- or post-copulation), at least to a degree. One might anticipate that *Mnais* females would prefer to mate with territorial males that have proved themselves in securing superior oviposition sites (Nomakuchi and Higashi 1985; Tsubaki et al. 2010), especially since the territorial male also protects them from further harassment while they are ovipositing. Moreover, it has also been noted that females mated by clear-winged males do not tend to oviposit without mating again (Nomakuchi and Higashi 1985), although the extent to which this is driven by female choice is unclear.

There is another remarkable feature of the polymorphism in *Mnais* that may reveal something of the underlying mechanisms that maintain it. Two related species of *Mnais,* namely *M. costalis* Selys, 1869 and *M. pruinosa* Selys, 1853, are distributed widely in Japan (Hayashi et al. 2004; Tsubaki and Okuyama 2016; Futahashi 2017). In regions where these species are allopatric, the males of both species exhibit the same orange-clear winged polymorphism. However, in many locations in central Japan where they co-occur the males of *M. costalis* have all evolved orange wings, while the males of *M. pruinosa* have all evolved clear wings (Tsubaki and Okuyama 2016). Since the two species show phenotypic differences when sympatric compared to when allopatric, we henceforth refer to these changes as “character displacement” (Pfennig and Pfennig 2009). When in sympatry, the orange-winged *M. costalis* males attempt to secure and defend territories just as they do in allopatry. However, the clear-winged *M. pruinosa* males in sympatry are more flexible in that they will frequently defend mating territories. Even here, however, the clear-winged males do not engage in courtship display and will flexibly behave as sneakers, suggesting that they have not lost their opportunistic behavior (Tsubaki and Okuyama 2016).

### Aims

The aims of our paper are two-fold. First, we present and explore a general game-theoretical model of territorial/sneaker interactions which we feel extends the existing game theoretical approaches to the general phenomenon of ARTs in two important ways: by allowing non-uniform interaction rates and considering female mate choice. Second, motivated by the *Mnais* system, we ask whether the same model can be used to understand why males tend to evolve monomorphism when two polymorphic species co-occur. In this case, explicit consideration of female choice is particularly important. Thus, while distinguishing characters may have arisen in males at least in part to reduce the frequency of needless contests with heterospecifics (Grether et al. 2009) (competitive interference), if females choose males based partly on the probability that they are conspecifics (reproductive interference), then it has the potential to cause runaway selection in which female mating preferences promote one male strategy over another, reinforcing selection on female mate preference (Lemmon 2009).

### Single species (allopatric) model

We consider a two-strategy game between territorial and sneaker males, both of which attempt to mate with females at oviposition sites. As usual, we make the “phenotypic gambit” (Grafen 1984) by exploring the evolutionary dynamics of male phenotypes under the assumption that “like begets like,” while trusting that the underlying genetics will enable these solutions to be reached. In the case of *Mnais*, this is readily justified: as Maynard Smith (1982) argued, when there are only two pure strategies then if the mixed strategy is stable, so is any genetic polymorphism.

Let males gain a mean payoff *V* (>0) from successfully fertilizing a female. Whenever a territorial male encounters a female in the presence of a sneaker, then we assume that territorial males display to the female at a cost *d* (≥0) and that they successfully fertilize the female on a proportion *q* (0 ≤ *q* ≤ 1) of such occasions. So, their average payoff is *V q* – *d*. Conversely, we assume that sneakers gain paternity by stealth on the remaining proportion (*1* – *q*) of occasions but do not pay a display cost, so their gain is *V* (*1* – *q*) on average. When two territorial males (whether neighbors, or an intruder opportunistically challenging a resident) contest a newly-encountered female then they both pay a cost of fighting *C*, but the victor also pays a cost to display to the female, so the mean payoff from this interaction is (*V* – *d*)/2 – *C*. Finally, two sneakers may fight to gain access to a female or suitable perch. Since sneaker-sneaker fights tend to be relatively aggressive when they occur (see, eg Nomakuchi et al. 1984) then to avoid adding too many parameters, we assume that the underlying cost of sneaker-sneaker interactions is the same as that between territorial individuals so that their average payoff in these interactions is *V*/2 – *C* (similar to the average payoff from territorial-territorial interactions, but without the display cost). Naturally, in practice the costs of contest and display will vary between interactions, but for sufficiently high number of interactions, selection would remain the same given that, for example, $\sum_{i=1}^{n}\left(\left(\frac{V_{i}}{2}\right)-C_{i}\right)/n=\left(\frac{\bar{V}}{2}\right)-\bar{C}$.

Not all pairwise interactions between males will be based on direct competition to mate with a female. For example, territorial males will fight with one another (without display) in the absence of a female to retain a territory, while sneaker males may fight with one another to obtain suitable perch sites from which to sneak copulations. We therefore assume that only a proportion *m* of male-male interactions involve competition for a female while the remaining (1 – *m*) of interactions simply take the form of jostling for a position with no immediate opportunity to mate with a female. Note that if male-male contests for territories happened more frequently earlier in the season as they are being established, then the parameter *m* would still capture the fact that females are not always present when male-male interactions occur. Under the above conditions, the overall payoff matrix $\left(\begin{array}{lll} W\left(T,T\right)\; & W\left(T,S\right)\;W\left(S,T\right) & W\left(S,S\right)\; \end{array}\right)\;$ is as follows:

**Table:** 

Payoff to player	Interacting with	
	T	S
T	(((V−d)/2)−C)m−C(1−m)	(Vq−d)m
S	V(1−q)m	$\left(\frac{V}{2}-C\right)m-C\left(1-m\right)$

While males may interact in the absence of a female, one might anticipate that males could occasionally mate with a female without engaging in a pairwise contest with any other male. In our Supplementary materials we show how one could modify the model to allow for this possibility, but as our model is already complicated enough (especially when two sympatric species interact) we focus on this simpler 2 × 2 version here.

How do we identify the evolutionary outcome when males with a given strategy gain a payoff dependent on the strategy of the male it is contesting? With uniform interaction rates, the selection dynamics of territorials and sneakers can be described by the replicator equation (Taylor and Jonker 1978; Hofbauer and Sigmund 1998). Thus, if *x* represents the frequency of territorial (T) males in the population, then:


$$\begin{array}{l} \dot{x}=x\left(1-x\right)\left(f_{T}-f_{S}\right) \end{array}$$

where $f_{T}$ and $f_{S}$ represent the mean payoff to a strategy per interaction to territorials and sneakers such that:


$$\begin{array}{l} \begin{array}{lll} & f_{T}=x\;W\left(T,T\right)+\left(1-x\right)W\left(T,S\right)\; & f_{S}=x\;W\left(S,T\right)+\left(1-x\right)\;W\left(S,S\right)\; \end{array} \end{array}$$

Let us now allow the probability of interaction between the two types of male to differ according to strategy, so that T males interact with other T males at a rate *r*_*TT*_, T males interact with S males at a rate *r*_*TS*_ (=*r*_*ST*_) and S males interact with S males at a rate *r*_*SS*_. Under these conditions we have:


$$\begin{array}{l} \begin{array}{lll} & f_{T}=\frac{x\;r_{TT}W\left(T,T\right)+\left(1-x\right)r_{TS}W\left(T,S\right)}{x\;r_{TT}+\left(1-x\right)r_{TS}}\; & f_{S}=\frac{x\;r_{TS}W\left(S,T\right)+\left(1-x\right)r_{SS}W\left(S,S\right)}{x\;r_{TS}+\left(1-x\right)r_{SS}}\; \end{array} \end{array}$$

Clearly, when *r*_*TT*_ = *r*_*TS*_ = *r*_*SS*_ the interaction rates cancel out and the denominator sums to 1, leaving us with the standard model as a special case. Taylor and Nowak (2006) fully characterized all two-strategy games with fixed payoffs and unequal interaction rates, noting that the conditions for the co-existence of the two strategies remain the same, the proportion of each strategy at equilibrium is dependent on interaction rates and that additional internal (polymorphic) equilibria are now possible for parameter combinations that would previously always result in one strategy dominating the other. How can we incorporate female choice in the model? Let us assume that the probability (*q*) of a territorial male fertilizing a female in a territorial-sneaker contest over access to a female depends on a combination of male-male competition (dictated by *q*_*M*_) and female preference (dictated by *q*_*F*_) such that:


$$\begin{array}{l} q=w\;q_{M}+\left(1-w\right)q_{F} \end{array}$$

where *w* is the weighting given to male competition compared to female choice (when *w* = 1 there is no female choice). Here *q*_*M*_ can be interpreted as the probability that the territorial male in a territorial-sneaker interaction will fertilize the female if males exclusively determine paternity (since *q* → *q*_*M*_ as *w* → 1). Similarly, *q*_*F*_ can be interpreted as the probability that the territorial male in a territorial-sneaker interaction will fertilize the female if females exclusively determine paternity (since *q* → *q*_*F*_ as *w* → 0). If a female prefers to mate with a territorial male, then *q *= *w q*_*M*_ + (1 – *w*), but if the female prefers to mate with a sneaker male, then *q* is reduced to *w q*_*M*_. At the population level, if a proportion *y* of females prefer to mate with territorial males, then on average *q *= *w q*_*M*_ + (1 – *w*) *y*. Substituting for *q* we can recast the male strategy payoffs as:

**Table:** 

Payoff to row player	Interacting with	
	T	S
T	(((V−d)/2)−C)m−C(1−m)	$\left\{V\left(w\;q_{M}+\left(1-w\right)y\right)-d\right\}m$
S	$V\left(1-\left(w\;q_{M}+\left(1-w\right)y\right)\right)m$	$\left(\frac{V}{2}-C\right)m-C\left(1-m\right)$

Just as we model male strategy evolution, we can model the (co-)evolution of female choice through replicator dynamics in which a proportion (*y*) of females prefer to mate with territorial males, and the remaining proportion (1 – *y*) prefer to mate with sneaker males. Let *b*_*T*_ represent the payoff to a female from mating with a territorial male and *b*_*S*_ represent the payoff to a female from mating with a sneaker male. Moreover, let $F_{Z}\left(X,Y\right)\;$ refer to the payoff gained by a female that prefers to mate with male strategy Z (∈ T,S) when male strategies X (∈ T,S) and Y (∈ T,S) compete to mate with the female. Finally, let $p_{XY}$ refer to the relative probability that the pairwise contest over a female is between male strategies X and Y. Under these conditions:


$$\begin{array}{l} \dot{y}=y\left(1-y\right)\left(g_{T}-g_{S}\right) \end{array}$$

where *g*_*T*_ and *g*_*S*_ are the mean payoffs per interaction to females that prefer territorials and sneakers, such that:


$$\begin{array}{l} \begin{array}{lll} & g_{T}=F_{T}\left(T,T\right)p_{TT}+F_{T}\left(T,S\right)p_{TS}+F_{T}\left(S,S\right)p_{SS}\; & g_{S}=F_{S}\left(T,T\right)p_{TT}+F_{S}\left(T,S\right)p_{TS}+F_{S}\left(S,S\right)p_{SS}\; \end{array} \end{array}$$

Clearly the female cannot act on its preference when two identical types of male compete to mate with it, so $F_{T}\left(T,T\right)p_{TT}=F_{S}\left(T,T\right)p_{TT}$ and $F_{T}\left(S,S\right)p_{SS}=F_{S}\left(S,S\right)p_{SS}$. Since these terms will cancel out when calculating the difference in payoffs per interaction $\left(g_{T}-g_{S}\right)$ then they do not need to be enumerated. Focusing on interactions between T and S males, the payoffs (per interaction) of territorial-preferring and sneaker-preferring females are:


$$\begin{array}{l} \begin{array}{lll} & F_{T\;}\left(T,S\right)=b_{T}\left(wq_{M}+\left(1-w\right)\right)+b_{S}(1-\left(wq_{M}+\left(1-w\right)\right)\; & F_{S\;}\left(T,S\right)=b_{T}wq_{M}+b_{S}\left(1-wq_{M}\right)\; \end{array} \end{array}$$

The probability that any given interactions is between a territorial and a sneaker (since *r*_*TT*_, *r*_*TS*_, *r*_*SS*_ refer to the overall rates of interaction rather than probabilities) is:


$$\begin{array}{l} p_{TS}=x\;\left(1-x\right)\;r_{TS}/\left(x^{2}r_{TT}+x\left(1-x\right)r_{TS}+{\left(1-x\right)}^{2}r_{SS}\right) \end{array}$$

Note the proportion of male-male interactions with a female present (*m*) cancels out since it has been assumed to be the same for all forms of interaction. Moreover, given that $\left(g_{T}-g_{S}\right)$ is reduceable to a constant ($F_{T}\left(T,S\right)-F_{S}\left(T,S\right))$ multiplied by a non-negative number ($p_{TS})$, then it is clear that the proportion of females that prefer territorials (*y*) will ultimately evolve towards 1 (if $b_{T}>b_{S})$ or 0 (if $b_{T}<b_{S})$. However, selection on *y* will approach zero as the proportion of males that are territorial approaches 0 or 1 because here T and S interactions arise so infrequently that female preference cannot be expressed.

Since the mean payoffs per interaction to territorial and sneaker males depends on how females choose between them, while the mean payoffs per interaction to territorial and sneaker-preferring females depends on the underlying frequency of territorials, the associated coupled ordinary differential equations representing the replicator dynamics are therefore:


$$\begin{array}{l} \begin{array}{lll} & \dot{x}=x\left(1-x\right)\left(f_{T}\left[y,\right]-f_{S}\left[y,\right]\right)\; & \dot{y}=y\left(1-y\right)\left(g_{T}\left[x,\right]-g_{S}\left[x,\right]\right)\; \end{array} \end{array}$$

### The two species (sympatric) model

In the first part of our analysis, we characterize the evolutionary dynamics of the above model with an aim of identifying the conditions for a stable polymorphism of territorial and sneaker males in the presence of unequal interaction rates and evolving female mate choice. In the second part of our paper, we seek to understand why two polymorphic species would lose their polymorphism and evolve distinct morphs when they come together. To do this, we further extended our model to represent the dynamics of male strategies and female preferences immediately following the secondary contact of two species.

Our analysis of changes in morph frequencies in sympatry immediately following secondary contact necessarily requires some simplifying assumptions. First, we assume that any cross-species hybrids are non-viable. Second, we assume that when the species initially make secondary contact, the males do not attempt to distinguish conspecific and heterospecific females (or conspecific and heterospecific males of the same morph), and neither do the females. Third, we further simplify the dynamics by assuming that the two species are free mixing. The above assumptions can be relaxed by allowing a degree of viability in crosses, by incorporating signal detection theory into discriminative decisions (Sherratt 2001) and by assuming species and morph-specific encounter rates ($r_{T1T1},r_{S1T2}\;$ etc) to reflect species segregation. However, it is not necessary to invoke these details to understand the processes we seek to highlight.

Let *ρ* be the relative frequency of the first species so that the second species has a relative frequency of 1-*ρ*. We now let *x*_*1*_ and *x*_*2*_ represent the proportion of territorial morphs in species 1 and 2 respectively, and *y*_*1*_ and *y*_*2*_ represent the proportion of species 1 and 2 females that prefer to mate with territorial males. Under the above conditions, the payoff to species 1 males of a given strategy interacting with males of a given strategy (whether species 1 or 2) is:

**Table:** 

Payoff to row male of species 1	Interacting with	
	T	S
T	$\left[\left(\frac{V-d}{2}-C\right)\rho +\left(\frac{-d}{2}-C\right)\left(1-\rho \right)\right]m-C\left(1-m\right)$	$\left\{V\left(w\;q_{M}+\left(1-w\right)y_{1}\right)-d\right\}m\;\rho -d\;m\left(1-\rho \right)$
S	$V\left(1-\left(w\;q_{M}+\left(1-w\right)y_{1}\right)\right)\rho \;m$	$\left[\left(\frac{V}{2}-C\right)\rho -C\left(1-\rho \right)\right]m-C\left(1-m\right)$

Note that the payoff W(T,T) assumes that the victorious males continue to court heterospecific females (which are encountered with probability 1 – *ρ*) but gain no benefit. The W(S,S) payoff is calculated on the basis that when sneakers compete for access to a female that turns out to be a heterospecific they pay a mean cost *–C*. The payoff matrix to males of species 2 is analogous:

**Table:** 

Payoff to row male of species 2	Interacting with	
	T	S
T	$\left[\left(\frac{V-d}{2}-C\right)\left(1-\rho \right)+\left(\frac{-d}{2}-C\right)\rho \right]m-C\left(1-m\right)$	$\left\{V\left(w\;q_{M}+\left(1-w\right)y_{2}\right)-d\right\}m\;\left(1-\rho \right)-d\;m\;\rho$
S	$V\left(1-\left(w\;q_{M}+\left(1-w\right)y_{2}\right)\right)\left(1-\rho \right)m$	$\left[\left(\frac{V}{2}-C\right)\left(1-\rho \right)-C\rho \right]m-C\left(1-m\right)$

Note that setting *ρ* to 1 renders the original model for species 1 and setting *ρ* to 0 renders the original model for species 2. The payoffs to the territorial-preferring and sneaker-preferring females arising from T and S interactions are as follows:

**Table:** 

Payoff to females	Prefer to mate with	
	T	S
Species 1	$p_{T1}b_{T}\left(wq_{M}+\left(1-w\right)\right)+p_{S1}b_{S}(1-\left(wq_{M}+\left(1-w\right)\right)$	$p_{T1}b_{T}wq_{M}+p_{S1}b_{S}\left(1-w\;q_{M}\right)$
Species 2	$p_{T2}b_{T}\left(wq_{M}+\left(1-w\right)\right)+p_{S2}b_{S}(1-\left(wq_{M}+\left(1-w\right)\right)$	$p_{T2}b_{T}wq_{M}+p_{S2}b_{S}\left(1-w\;q_{M}\right)$

where *p*_*T1*_ is the probability that a territorial male is species 1 (= *ρ x*_*1*_/(*ρ x*_*1*_ + (1 - *ρ*) × _*2*_)) and *p*_*S1*_ is the probability that a sneaker male is species 1 (= *ρ (1-x*_*1*_)/(*ρ (1- x*_*1*_) + *(1-ρ)* (1- *x*_*2*_)). The probability that a territorial male is species 2 (*p*_*T2*_) is the complement, ie (1-*p*_*T1*_), as is the probability that a sneaker male is species 2 (*p*_*S2*_), ie (= 1- *p*_*S1*_). Again, these payoffs reduce to the respective single species model for *ρ *= 1 and *ρ *= 0. Assuming the species are free mixing, the relative probabilities of a male-male contest over a female being between a T and S interaction is:


$$\begin{array}{l} p_{TS}=v\left(1-v\right)r_{TS}/\;\left(v^{2}r_{TT}+v\left(1-v\right)r_{TS}+{\left(1-v\right)}^{2}r_{SS}\right) \end{array}$$

where:


$$\begin{array}{l} v=x_{1}\rho +x_{2}\left(1-\rho \right) \end{array}$$

Combining the male strategies and female preferences of each species, the coupled differential equations representing their replicator dynamics are therefore:


$$\begin{array}{l} \begin{array}{lllll} & \overset{}{{\dot{x}}_{1}}=x_{1}\;\left(1-x_{1}\right)\left(f_{T1}\left[y_{1},\right]-f_{S1}\left[y_{1},\;\right]\right)\; & \overset{}{{\dot{x}}_{2}}=x_{2}\;\left(1-x_{2}\right)\left(f_{T2}\left[y_{2},\right]-f_{S2}\left[y_{2},\right]\right)\; & \overset{}{{\dot{y}}_{1}}=y_{1}\;\left(1-y_{1}\right)\left(g_{T1}\left[x_{1},\;x_{2},\;\right]-g_{S1}\left[x_{1},\;x_{2},\;\right]\right)\; & \overset{}{{\dot{y}}_{2}}=y_{2}\;\left(1-y_{2}\right)\left(g_{T2}\left[x_{1},\;x_{2},\;\right]-g_{S2}\left[x_{1},\;x_{2},\;\right]\right)\; \end{array} \end{array}$$

## Results

Where possible, we have analytically elucidated the conditions under which different qualitative outcomes (eg domination by one strategy, stable polymorphism) will arise. Specific examples of the model were implemented by numerically integrating the coupled ordinary differential equations used to represent the replicator dynamics over a sufficient time (typically to *t* = 10,000) for a long-term outcome to be achieved. Parametric numerical integration was used to elucidate the effects of simultaneously changing several parameters on the nature of the outcome and derive the region plots. Without loss of generality, we can set the mean reward to a male from successfully mating with a female (*V*) to 1 and express the associated mean fighting and display costs (*C* and *d*) on this scale. Similarly, since it is only the relative interaction rates that matter to the evolutionary dynamics, we have set *r*_*TS*_ to 1 and scale *r*_*TT*_ and *r*_*SS*_ accordingly (0 < *r*_*TT*_, *r*_*SS*_ < ∞).

### Single species (allopatric) model

If *b*_*T*_ = *b*_*S*_ (the benefits to females from mating with a territorial are equal to those of females mating with a sneaker) then *y* (the proportion of females preferring territorial males) will not change under selection and *q* (the proportion of territorial vs. sneaker interactions resulting in paternity by territorials) will consequently be a constant. The evolutionary dynamics for all 2 × 2 games with fixed payoffs under any combination of strategy-dependent interaction rates have been elucidated by Taylor and Nowak (2006). Thus, there are regions under which (a) territorials dominate sneakers or *vice versa* (with non-uniform interaction rates, two interior equilibria are possible in sub-regions of this parameter space—one stable and one unstable; which attractor is converged on depends on the initial conditions), (b) regions of bi-stable dynamics in which either territorials or sneakers dominate (with the outcome again dependent on the initial conditions) and (c) regions in which a stable polymorphism of territorial and sneaker males arise. The combination of conditions required for a stable polymorphism are identical to that with uniform interaction rates, although the precise frequency of territorials at equilibrium is dependent on the rates of interaction. For constant *q* then the conditions for polymorphism readily reduce to:


$$\begin{array}{l} C/m>max[\left(2\;q-d-1\right)/2,\;\;\left(1+2\;d-2q\right)/2] \end{array}$$

Thus, the greater the cost of fighting (the higher *C*) and the more frequent this fighting occurs in the absence of a female (the lower *m*) the more likely a polymorphism will arise. This is readily understood in qualitative terms since increasing *C* and decreasing *m* will both lower W[T,T] and W[S,S] compared to W[T,S] and W[S,T], making the underlying inequalities for polymorphism more likely to be fulfilled.

**Fig. 2.**
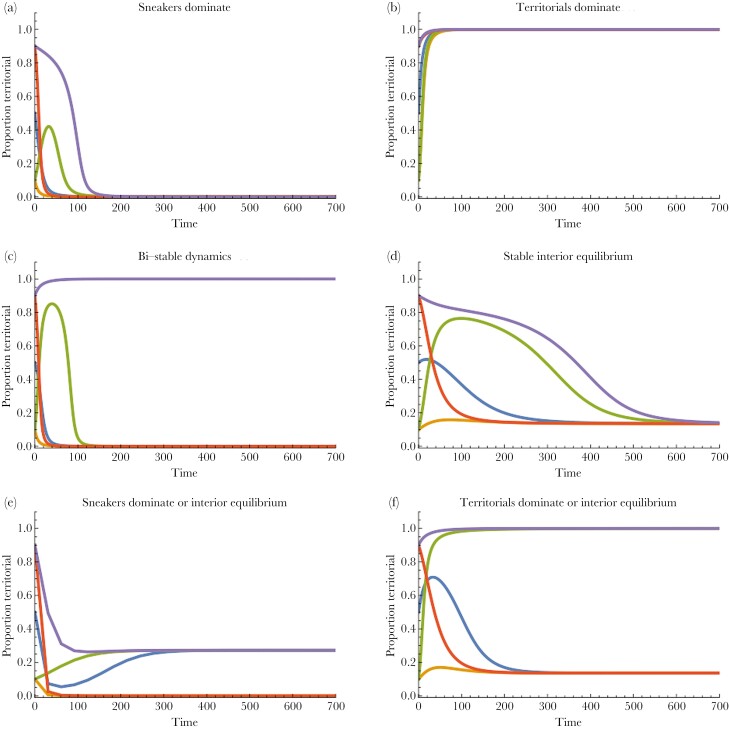
(a–f) Six qualitatively different outcomes of the one-species (allopatric) game under replicator dynamics when territorial and sneaker males interact with one another at different relative rates, and the preference of females for territorial over sneaker males is also free to evolve. The dynamical plots were derived under five different starting conditions namely *x*[0] = 0.5, *y*[0] = 0.5 (blue); *x*[0] = 0.1, *y*[0] = 0.1 (orange); *x*[0] = 0.1, *y*[0] = 0.9 (green); *x*[0] = 0.9, *y*[0] = 0.1 (red); *x*[0] = 0.9, *y*[0] = 0.9 (purple). Here we have assumed *C* = 0.1, *d* = 0.05, *m* = 0.4, *r*_*TT*_ = 0.5, *r*_*SS*_ = 0.1, *b*_*T*_* *= 0.9, *b*_*S*_ = 1 except where indicated. Sneakers dominate: *w* = 0.4, *q*_*M*_* *= 0.3; Territorials dominate: *w* = 0.95, *q*_*M*_ = 0.95; Bi-stable dynamics: *w* = 0.1, *q*_*M*_ = 0.8; Stable interior equilibrium: *w* = 0.8, *q*_*M*_ = 0.6; Sneakers dominate or interior equilibrium: *w* = 0.6, *q*_*M*_ = 0.2, *b*_*T*_* *= 1, *b*_*S*_ = 0.9; Territorials dominate or interior equilibrium: *w* = 0.6, *q*_*M*_* *= 0.8. Note that a given male strategy can dominate even if females gain more from choosing the alternate strategy (so that sneakers can dominate even if *b*_*T*_* *> *b*_*S*_ as in Fig. 2e, or territorials can dominate even if *b*_*T*_ < *b*_*S*_ as in Fig. 2f) simply because female preference is not sufficient to tip the balance. Figure S1a–f shows the corresponding vector plots for the instantaneous rates of change of the proportion of males that are territorial (*x*) and the proportion of females that prefer territorial males (*y*) under the same combinations of parameter values.

More generally, when *b*_*T*_ ≠ *b*_*S*_ (and 0 < *x*[0] < 1, 0 < *y*[0] < 1, *w* < 1) then the mean proportion of females that prefer territorial males (*y*), and hence *q*, will co-evolve with the frequency of territorial males. Under these more general conditions the same qualitative outcomes highlighted by Taylor and Nowak (2006) arise (see Fig. 2a–f and Fig. S1a–f). As Taylor and Nowak (2006) had previously observed, when two stable equilibria are possible in certain regions of parameter space, then the starting conditions (in this case both *x*[0] and *y*[0]) can affect the stable state that is ultimately converged on.

**Fig. 3.**
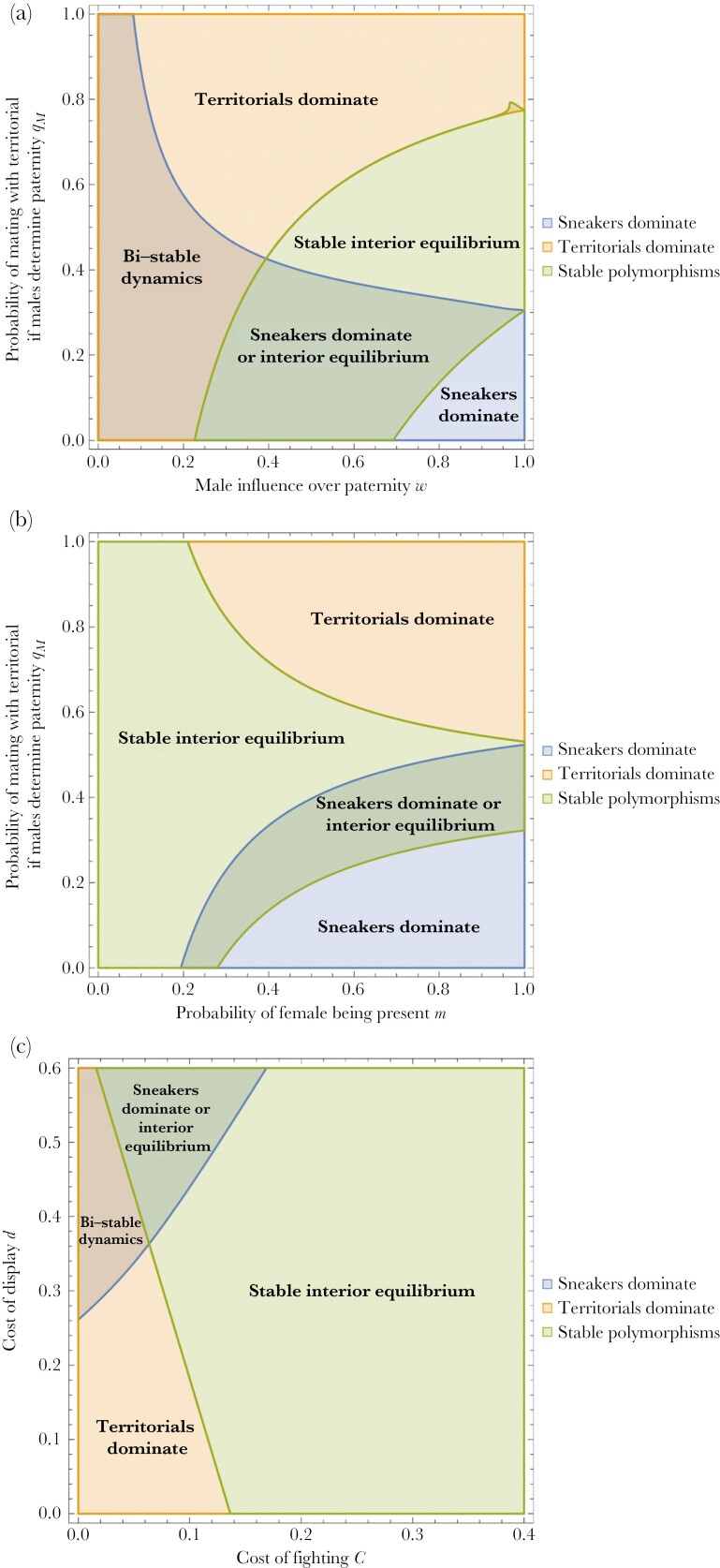
(a–c) Region plots of the outcome of dynamics in *x*[t] after *t *= 100,000. Five separate starting conditions for *x*[0] and *y*[0] were assumed namely: *x*[0] = 0.5, *y*[0] = 0.5; *x*[0] = 0.1, *y*[0] = 0.1; *x*[0] = 0.1, *y*[0] = 0.9; *x*[0] = 0.9, *y*[0] = 0.1; *x*[0] = 0.9, *y*[0] = 0.9. When the final frequency of territorials was above 0.999 then it was considered dominant, and when it was consistently below 0.001 then sneakers were considered dominant. Any final frequency in between these levels reflects a stable interior equilibrium. As shown in Fig. 2a–f, for any given set of parameter values the outcome could potentially depend on the start conditions, so that under one set of conditions we see one outcome arising (eg territorials dominate) and another set of start conditions we see another outcome arising (eg a stable interior equilibrium). Default parameters values (before varying pairs of parameters in each graph): *C* = 0.1, *d* = 0.05, *m *= 0.4, *w* = 0.8, *q*_*M*_* *= 0.8, *r*_*TT*_ = 0.5, *r*_*SS*_ = 0.1, *b*_*T*_ = 1; *b*_*S*_* *= 0.9. Under the conditions assumed, there are wide regions of parameter space (depicted in light green) where a stable interior equilibrium (ie polymorphism in territorials and sneakers) would consistently arise under all considered starting values of x[0] and y[0]. See Figure S2a–c for region plots under identical conditions except *b*_*T*_ = 0.9; *b*_*S*_* *= 1 (so that females will evolve a preference towards mating with sneaker males rather than territorial males).

The properties of the model are relatively intuitive. For example, as Fig. 3a illustrates, territorial males will tend to dominate if they compete well with sneakers for fertilizations (*q*_*M*_ is high) and males have a high influence over paternity (*w* is high). Conversely, sneaker males will dominate the population when *q*_*M*_ is low and males have a high influence over paternity. When females have a strong influence on paternity (*w* low) then either territorial males or sneaker males can evolve to fixation, depending on the starting conditions, with stable interior (polymorphic) equilibria evolving for combinations in between. Likewise, as Figure 3b shows, when fights commonly occur in the absence of a female (*m* low) then there is a broad range of conditions under which polymorphisms are favored (for an explanation, see above). Finally, as Fig. 3c shows, territorials dominate when the cost of fighting and cost of display are low. When the cost of display to females is high but the costs of fighting are low then sneakers are favored. When both the cost of display and fighting is high then a stable polymorphism will arise for the reasons given above.

### Two species (sympatric) model

**Fig. 4.**
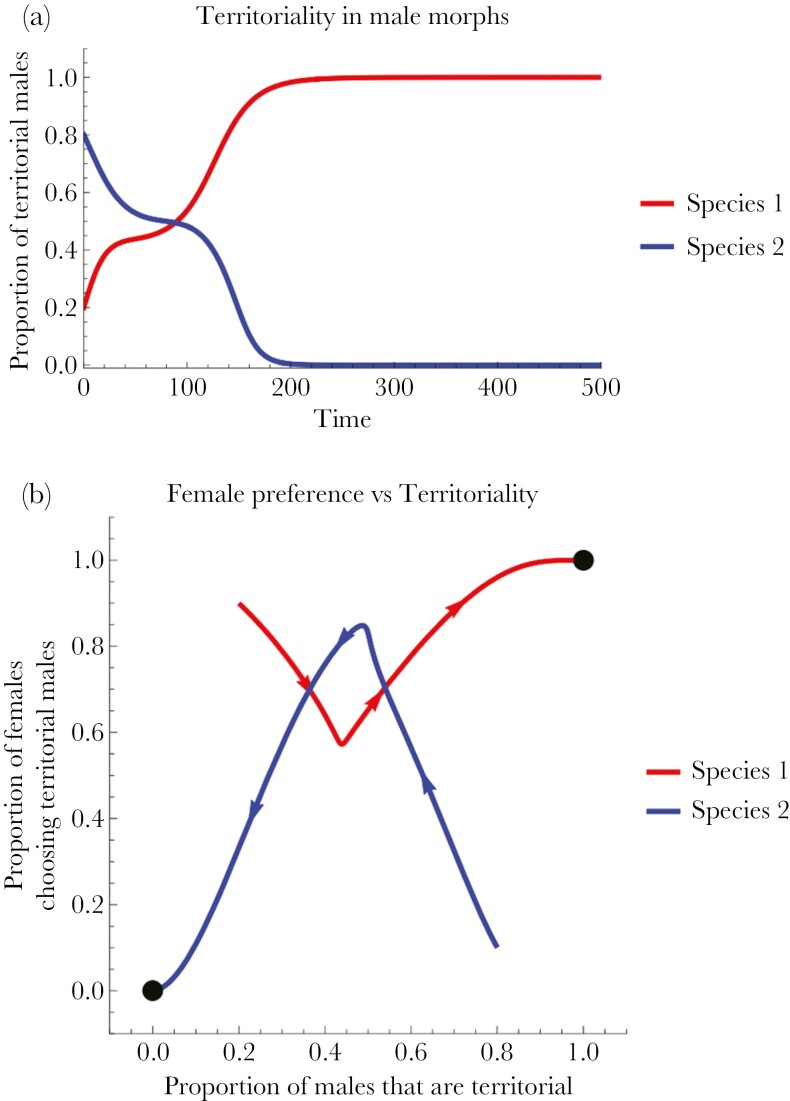
(a, b) The evolution of alternative male mating strategies and female preference when two species come into secondary contact. Here the females of the two species, and the males of the same morph of the two species, are assumed to be indistinguishable. Parameters: *C* = 0.1, *d* = 0.05, *m = 0.4; w *= 0.6, *q*_*M*_* *= 0.6, *r*_*TT*_* *= 0.5; *r*_*SS*_* *= 0.1, *b*_*T*_ =1, *b*_*S*_ = 0.9, *ρ* = 0.7, starting conditions: *x*_*1*_[0] = 0.2, *y*_*1*_[0] = 0.9, *x*_*2*_[0] = 0.8, *y*_*2*_[0] = 0.1. These conditions would produce a stable polymorphism in each species were they allopatric. However, when the species are sympatric, species 1 females evolve to prefer territorial males through which they gain the highest fitness. By contrast, the rarer species 2 females evolve a preference for sneaker males which they would not otherwise favor except that they need to discriminate conspecifics from heterospecifics. Male morphs in each species evolve to match these evolved preferences.

**Fig. 5.**
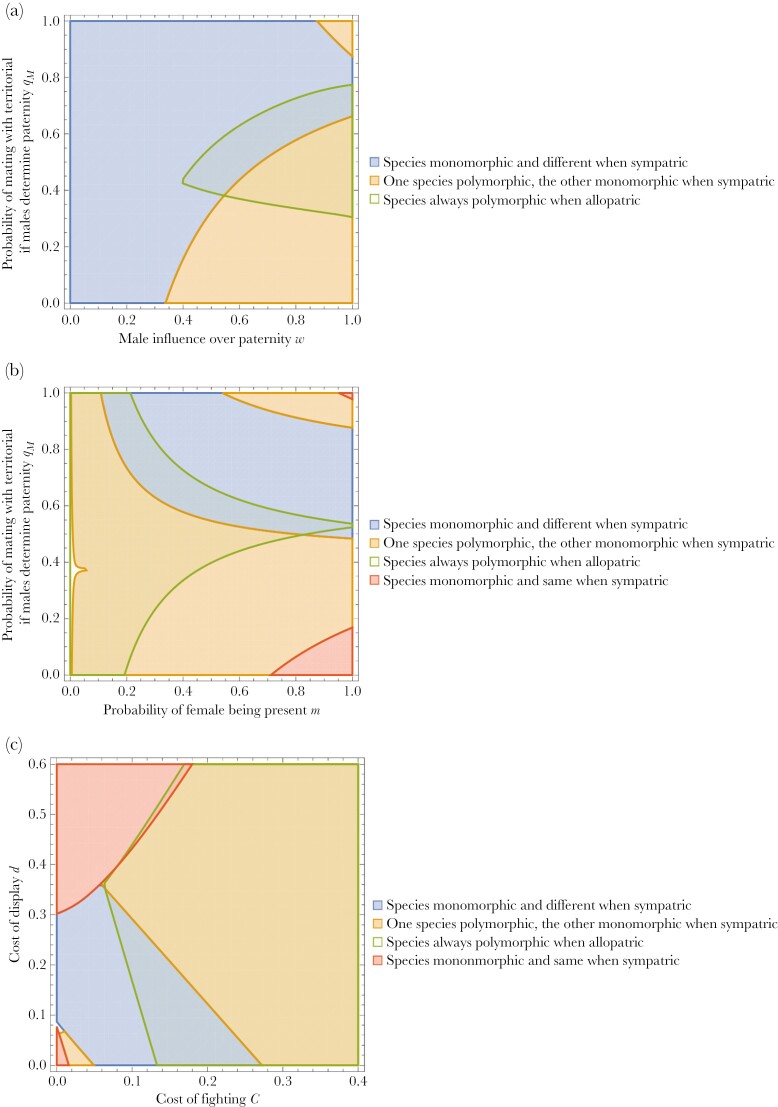
(a–c) The outcome after *t *= 10,000 when two species make secondary contact in sympatry compared to the outcome had they remained allopatric. Parameter values were the same as Fig. 3a–c with *ρ *= 0.7. The starting conditions were *x*_*1*_[0] = 0.5, *x*_*2*_[0] = 0.5, *y*_*1*_[0] = 0.9, *y*_*2*_[0] = 0.9 (reflecting female preference for territorials when allopatric). When sympatric the males two species can evolve to be monomorphic and distinct (blue), monomorphic and identical (red) or one species can evolve polymorphism while the other evolves to be monomorphic (brown). Overlaid in green are the conditions under which the species is always polymorphic when alone (allopatric); see Fig. 3a–c. There are wide ranges of conditions in each plot under which male polymorphism will arise in allopatry, while distinct monomorphism will evolve in sympatry (ie green overlaid by blue).

Figure 4a and b shows a single implementation of the model in which the male reproductive strategies and female mating preference separately evolve in each species. The parameter conditions represent a case in which each of the two species would have been polymorphic when allopatric, but now we see that the territorial morph evolves to fixation in one species and the sneaker morph evolves to fixation in the other. This outcome arises despite the fact that one type of conspecific male (here, territorials) is more profitable for a female to mate with than another. Figure 5a–c shows a broader region plot under the same conditions as considered in Fig. 3a–c, this time following a period of secondary contact between two species. Once again, we see that when sympatric, one species can become dominated by sneakers and one species becomes dominated by territorials (blue regions). This is, however, not the only endpoint. Under some conditions, one species will evolve a monomorphism and the other species will retain its polymorphism (orange regions, see Fig. 5a–c). Similarly, if there is no polymorphism in either species when allopatric, then there may continue to be no polymorphism when sympatric (red regions).

Several questions arise. First and foremost, is the evolution of distinguishing forms of males entirely a product of female preference or is it also a consequence of selection on males to avoid heterospecifics in competition? Note that while a low *w* (ie a strong influence of female choice on the outcome of mating) strongly favors distinct male monomorphism when the species are sympatric, there is also a narrower range of conditions under which the same effect arises when *w* = 1 (Fig. 5a) where females do not express their preference. Thus, even when male competition alone dictates paternity, then the cost of heterospecific fighting, coupled with misdirected mating attempts (see Discussion) can sometimes drive the males of the two species to evolve distinct phenotypes.

**Fig. 6.**
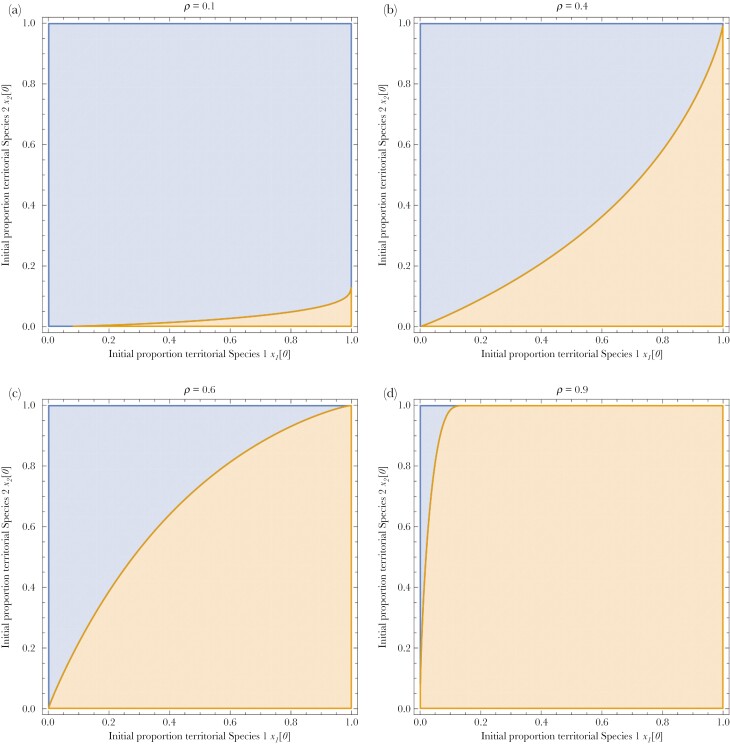
(a–d) How the frequency of species 1 (*ρ*, shown in separate graphs) and the initial frequency of males of each species that are orange-winged territorials (*x*_*1*_[0] and *x*_*2*_[0]) affect the species that ultimately evolves orange-winged territorial males when the species evolve distinct morphs in sympatry. The evolved strategy of species 1 is shown (blue - clear, orange - territorial) with species 2 evolving the alternate strategy. Parameter values: *C *= 0.1, *d* = 0.05, *m* = 0.4, *w* = 0.6, *q*_*M*_ = 0.6, *r*_*TT*_ = 0.5; *r*_*SS*_* *= 0.1, *b*_*T*_ =1, *b*_*S*_ = 0.9. Here we set *y*_*1*_[0] = 0.9, *y*_*2*_[0] = 0.9 to reflect the case that when in allopatry females will tend to evolve a preference for territorial males (since *b*_*T*_ > *b*_*S*_). When a species is relatively common then its males will tend to evolve the form with which it is most profitable for conspecific females to mate with, while the relatively rare species will evolve the less profitable male type to reduce the rate at which it mistakenly mates with heterospecifics. However, when the two species are at similar frequency, the species with the greatest majority of one type will tend to evolve that type. When the inequality is reversed such that *b*_*T*_ < *b*_*S*_ then the common species will tend to evolve preference for sneakers, while the rarer type will tend to evolve preference for the less profitable male type to enhance discrimination.

Second, one might wonder what asymmetries determine which species evolves the territorial (orange-winged) morph and which one evolves the sneaker (clear-winged) morph. From comparing the female payoff functions when territorial and sneaker males compete for a female, it is readily shown that a female of species 1 would gain higher fitness from preferring to mate with a territorial male if:


$$\begin{array}{l} \frac{b_{S}}{b_{T}}<\frac{x_{1}\left(x_{2}+\rho \left(x_{1}-x_{2}\right)-1\right)}{\left(x_{1}-1\right)\left(x_{2}+\rho \left(x_{1}-x_{2}\right)\right)} \end{array}$$

and would prefer to mate with a sneaker male if this inequality were reversed. An analogous inequality can be derived for species 2. Thus, the optimal female preference of each species at any given point in time is dependent not just on the relative profitability of mating with a territorial or sneaker male assuming it were a conspecific (*b*_*S*_ and *b*_*T*_), but also on the relative likelihood that the male type is from their own species, which is dictated by *x*_*1*_, *x*_*2*_ and ρ. Figure 6a–d shows that males of the more common species will evolve the male form that is most profitable for females to mate with (in this case territorials) in allopatry. Conversely males of the rarer species evolve the male form that is less profitable for females to mate were it a conspecific. The reason for this difference in evolved preference is that females of the rarer species are more liable to mate with heterospecifics than females of the more common species, and so will face stronger selection to avoid mistakes (see Discussion). When the species are at similar overall frequencies when they make secondary contact (ρ≈0.5), then the frequencies of each male strategy in each species (*x*_*1*_ and *x*_*2*_) will influence the outcome, with females of each species preferring the male type that is relatively more common in their species compared to the heterospecific.

## Discussion

Two unstated assumptions of the two-strategy territorial-sneaker games previously invoked to explain the evolution and maintenance of alternative reproductive tactics (Tainaka et al. 2007; Tanaka et al. 2009) are that: (1) males interact with one another at a rate that is independent of their strategy, and (2) female choice, if it arises, is not subject to selection. Yet if territory holders are dispersed over available territories, and sneakers remain at the edges of territories seeking to obtain a mate with females by stealth, then territorial-sneaker interactions over females may be disproportionately more common than any other form of male-male interaction (Nomakuchi et al. 1984; Ota and Kohda 2015). Likewise, females are not passive observers of male competition and may strategically seek to control the type of male they mate with in a manner that varies with the prevailing conditions (Jennions and Petrie 1997). By extending the familiar territorial-sneaker game to allow for the possibility unequal interaction rates and evolving female choice (while allowing for the former to be equal and the latter to be fixed as special cases), we hope that we have broadened the applicability of these two-strategy games.

Our analysis of the single species (allopatric) system confirms that there is a broad range of plausible conditions under which a polymorphism of territorials and sneakers will evolve, with the solution representing a mixed evolutionarily stable strategy (ESS) (Maynard Smith 1982). For the 2 × 2 game involving fixed payoffs, then we require *W*(T,S) > *W*(S,S) and *W*(S,T) > *W*(T,T) for the male strategies to evolve towards a balanced equilibrium. One can readily envisage conditions under which W(T,S) > W(S,S) in our case example of *Mnais* damselflies, since clear-winged males tend to retreat when challenged by orange-winged males and yet clear-winged males also fight aggressively with other clear-winged males (Nomakuchi et al. 1984; Hooper et al. 2006). Likewise, the inequality *W*(S,T) > *W*(T,T) appears entirely plausible given the fact that territorials pay significant costs to establish and defend territories. Indeed, orange-winged males tend to deplete their fat reserves more rapidly than clear-winged males as they age (Plaistow and Tsubaki 2000), which likely reflects the costs of territory maintenance. Both inequalities will be further reinforced if fighting frequently occurs in the absence of a female (low *m*), because then the fighting costs of territorials against territorials and sneakers against sneakers accumulate even if there is no immediate reward.

Male-limited color polymorphism is a relatively rare phenomenon in damselflies (Rivas-Torres et al. 2019) and given the range of plausible conditions under which it arises in our mathematical model, one might wonder why it is not more prevalent. In many cases territorial and non-territorial males may be present within a population but go largely unrecognized because these males do not vary in color (Poethke and Kaiser 1987). By contrast, if there is selection to have a colorful display when territorial and yet individuals are also selected to be relatively inconspicuous when acting as a sneaker, then there may be differential selection on color depending on the individual’s strategy. In a formal mathematical model Plaistow et al. (2004) showed that pure strategies are most likely to evolve when the costs and/or limits of plasticity are high, and mating skew is high (which they quantified as the ratio of males to available territories). We view our model as complementary to Plaistow et al. (2004), in that we characterized the conditions under which a mixture of pure strategies is possible in allopatry, while they sought to elucidate the conditions that favor the evolution of conditional versus pure strategies.

Despite offering more transparency and analytical insight than a parameter-heavy, individual-based simulation, our model, as with all such strategic models, remains relatively abstract (see McNamara 2013) even with regard to the specific case of *Mnais* damselflies that motivated our work. In particular, we made no distinction in our model between orange-winged males that were successfully holding territories and those that were without territories, implicitly assuming that *W*(T,T) represents the average payoff from all such individuals. Another intriguing aspect of the biology of *Mnais* is that clear-winged males appear to live several days longer than orange-winged territorial males on average, both in the laboratory and in the field, with the frequency of clear-winged males increasing in the field as the season progresses (Tsubaki et al. 1997). Our model has not accounted for this intriguing difference in average adult longevity. However, Smallegange and Johansson (2014) have shown how one could combine game theory and demography by incorporating strategy-specific maturation rates into the differential equations used to represent their evolutionary dynamics. In our case, the differences in mean longevity have only been reported in adults and are of the order of a few days, but in other applications (such as salmonid fishes, where jacks return to their breeding grounds at least one breeding season before hooknoses, Watters 2005) these details may be very important. Finally, while replicator equations represent the most common way of representing evolutionary dynamics (Hofbauer and Sigmund 1998) there are alternative candidate models that might be considered, particularly for asymmetrical games in which players have different strategy sets and payoffs. For example, when discussing the asymmetric “battle of the sexes” model by Schuster and Sigmund (1981), Maynard Smith (1982) expressed “room for doubt” and advocated normalizing the rate equation by dividing by the mean payoff. We have not explored the evolutionary dynamics under this revised form or any other alternative form, but there is no reason to believe that our qualitative insights would differ.

The first aim of our modeling study was to understand how and why male ARTs were maintained when strategies do not interact at rates proportional to their densities and females exhibit mate choice. Our second aim was more ambitious: we sought to use the same model to help understand why the polymorphic males of two related species become monomorphic (and distinct) when in sympatry. Heterospecific interactions clearly play a role here, but elucidating candidate mechanisms requires formal modeling.

The contrasting patterns of the differentiability of sympatric *Mnais* species based on mitochondrial and nuclear genetic markers indicate that the species have been able to introgress in the recent past (Hayashi et al. 2005).

It has long been appreciated that if hybrids are less viable, then when two species come into secondary contact there will be selection to reduce the frequency of competitive and/or reproductive interactions between them (eg Hoskin et al. 2005). Tsubaki and Okuyama (2016) report unpublished data which suggests that when in sympatry male *Mnais* rarely respond to heterospecific females, although they also note that interspecific copulations are not entirely absent. Naturally these observations will have been made after a long period of co-existence in which the discriminative abilities of both males and females will have experienced strong selection (Tynkkynen et al. 2004). In the same way, after a long period of sympatry, we would expect to find that males are able to use wing color to distinguish conspecific from heterospecific males.

Character displacement in wing color pattern has already been reported in damselflies, notably differences in color patterns between congeneric *Calopteryx* species (Waage 1979; Tynkkynen et al. 2004) and congeneric *Hetaerina* species (Anderson and Grether 2010). The case of *Mnais* involves a complementary loss of one of the discrete male reproductive tactics in each species when in sympatry, although a degree of habitat segregation may also be involved (Okuyama et al. 2013; Tsubaki and Samejima 2016). *Mnais* may be a prime candidate for character displacement because, as Rice and Pfennig (2007) suggest, species with a mating polymorphism may be more prone to undergo phenotypic changes when sympatric. Indeed, Pfennig et al. (2006) reported that when two species of spadefoot toad (*Spea bombifrons* and *S. multiplicata*) occurred alone in the field, they produced similar proportions of omnivore and carnivore tadpoles, but when together *S. bombifrons* produced mostly carnivores and *S. multiplicata* produced mostly omnivores.

One of the questions commonly asked about character displacement is whether it is mediated by reproductive interference (ie it reflects selection against maladaptive hybridization) or agonistic interference (ie it reflects selection against inter-specific aggression) although the explanations are not mutually exclusive (Grether et al. 2009). To address the above question, Okamoto and Grether (2013) developed and explored a detailed individual-based model, confirming the potential for agonistic character displacement (ACD) to drive differences between species, but also noting that reproductive character displacement (RCD) sets the pace of divergence when the same traits are targets of both competitor recognition and mate recognition. Here we have presented a simpler model in explicit game theoretical terms, which confirms that selection for monomorphism in sympatry can arise even in the absence of female mate choice, albeit in a narrower range of parameter space. Note that removing female choice in our model is not equivalent to removing all reproductive interference because while males can needlessly fight with heterospecific males over a conspecific female, they may also mistakenly fight (and even display) with a conspecific male to mate with a heterospecific female.

As noted above, one of the primary (but not exclusive) drivers of character displacement in our model was female mate choice. To date there is no direct evidence of female mate choice for territorials over sneakers (or *vice versa*) in *Mnais*, because it has proved challenging to test experimentally. Of course, absence of evidence does not constitute evidence of absence (Córdoba-Aguilar et al. 2015). Siva-Jothy (1999) inferred that females of the Caloptergygid *Calopteryx splendens* choose males in part on their wing pigmentation which is displayed prominently to females during courtship (see also Córdoba-Aguilar 2002). Moreover, Tsubaki et al. (2010) found that orange-winged males of *Mnais costalis* with higher thorax temperatures not only showed more intensive courtship display than other members of the same morph, but also were more likely to copulate with females. They proposed that the disproportionate success of hotter males was a reflection of female choice. Even if direct female choice did not arise, then one might expect a female to be able to change the rate at which it interacts with certain types of male simply by changing its site preferences.

We noted earlier that in a well-mixed population the probability of any orange-winged male being species 1 was *ρ x*_*1*_/(*ρ x*_*1*_ + (1 - *ρ*) *x*_*2*_) while the probability of a sneaker male being species 1 was *ρ (1-x*_*1*_)/(*ρ (1- x*_*1*_) + *(1-ρ)* (1- *x*_*2*_)). So, for example if *ρ* *= *0.01, *x*_*1*_ = 0.52, *x*_*2*_ = 0.72 then a territorial has only a 0.007 probability of being species 1, but a sneaker has a probability of 0.017 of being species 1, which is more than double the probability. By contrast the probability of a territorial and sneaker being species 2 is 0.993 and 0.983 respectively. Females of the rare species will therefore face stronger selection on their mating preferences, than females of the common species (Hoskin et al. 2005; Lemmon 2009). In this instance, all else being equal, females of rarer species 1 that prefer to mate with sneakers in territorial-sneaker contests will leave more offspring than conspecific females that prefer to mate with territorials. As selection on female preferences develops, then so too will the payoffs to territorial and sneaker males, causing a concomitant increase in sneakers in species 1 and an increase in territorials in species 2. The result is a powerful feedback loop, analogous to Fisherian “runaway selection” (Fisher 1930) in which female choice selects for male strategies which further reinforces selection on female choice. Given that Okamoto and Grether (2013) allowed female mate preference to evolve, and mating with a heterospecific was assumed costly, then the same runaway process may also readily explain why RCD tended to dominate the evolution of the species recognition traits in their model.

The fact that females of the rarer species face stronger selection to change their mating preference, and that they may typically end up preferring a male morph that is not favored by the more common species (see Fig. 6a–d) has important implications. *M. costalis* is allopatric in the north of Japan while *M. pruinosa* is allopatric in the south where it is warmer (Futahashi 2017). It is reasonable to suppose that the two species are making secondary contact as *M. pruinosa* moves northwards, following the rising spring and summer temperatures since the end of the last glacial maximum (approximately 10,000 years BCE) and more recent anthropogenic climate change (Hickling et al. 2005; Nakanishi et al. 2020). If true, this would result in *M. pruinosa* typically being the rarer species upon secondary contact, which would imply that territorials are, all else being equal, the preferred morph yet *M. pruinosa* females switch their preference to sneakers to increase their chances of mating with a conspecific.

Finally, it is important to note that further evolution is possible in the *Mnais* system. Indeed, it may well be ongoing. In particular, in some populations in western Japan where the two *Mnais* species are believed to have been sympatric for the longest period, males of both species have regained their polymorphism (Futahashi 2017). Here male *M. pruinosa* (which are clear-winged in recent sympatry) have evolved a distinct orange and brown winged territorial morph (see Fig. S4), while male *M. costalis* (which were orange-winged in recent sympatry) has evolved a distinct pale orange sneaker morph (Futahashi 2017). Collectively, this suggests additional character displacement based on novel traits, rather than simply loss of existing ones. It may appear that the *Mnais* system has been dreamt up by game theorists, but the species complex offers an important opportunity to understand how closely related species can co-exist and how phenotypic diversity is generated and lost in natural populations.

## Supplementary Material

araf002_suppl_Supplementary_Figures_S1-S4

araf002_suppl_Supplementary_Materials

## Data Availability

We provide R code to implement the replicator-dynamics in the one- and two- species models by numerically integrating the associated coupled ordinary differential equations.
