# Supplementary material for: Territorial-sneaker games with non-uniform interactions and female mate choice
Source: Behav Ecol. 2025 Jan 17;36(2):araf002. doi: 10.1093/beheco/araf002 (PMC11786120; doi:10.1093/beheco/araf002)
Supplement: araf002_suppl_Supplementary_Figures_S1-S4 [file araf002_suppl_supplementary_figures_s1-s4.docx]

**Supplementary Information**

Territorial-sneaker games with non-uniform interactions

and female mate choice

Sherratt TN, Beatty CD, Dewan I, Di Iorio K, Finkelstein I,

Loeffler-Henry K, Miller M, Para F, Raposo M, Sherratt F.

**Figure S1a-f**

**Figure S1a-f.** Vector plots of the instantaneous rates of change in the proportion of males that are territorial (*x*) and the proportion of females preferring territorial males (*y*) from different starting conditions in the single species model. The direction and colour of the vectors reflect the nature and intensity of selection respectively. The red dots show the internal stable equilibria. The specific values of parameters used to derive these plots were the same as **Figure 2a-f** in the main text. Note that female preference will not evolve further when one male strategy dominates, because under these conditions, contests between territorials and sneakers over a female will not arise.

**Figure S2a-c**

**Figure S2a-c.** Region plots of the qualitative outcome of dynamics in *x*[t] after *t* = 10,000 under the starting conditions and parameter values specified in **Figure 3a-c,** this time with *b_T_* = 0.9, *b_S_* = 1 such that females will evolve a preference towards mating with sneaker males.

**Figure S3a-c**

**Figure S3a-c.** The outcome after *t* = 10,000 when two species make secondary contact compared to the outcome had they remained allopatric. Parameter values were the same as **Figure 5a-c** yet with *b_T_* = 0.9, *b_S_* = 1. The starting conditions for the two species case were *x_1_*[0]= 0.5, *x_2_*[0] = 0.5, *y_1_*[0]= 0.1, *y_2_*[0] = 0.1 (reflecting female preference for sneaker males when allopatric). Overlaid in green are the conditions under which the species is always polymorphic when alone (allopatric); see **Figure S2a-c**. There are wide ranges of conditions under which male polymorphism will arise in allopatry, while distinct monomorphism will evolve in sympatry (green overlaid by blue).

**Figure S4**

**
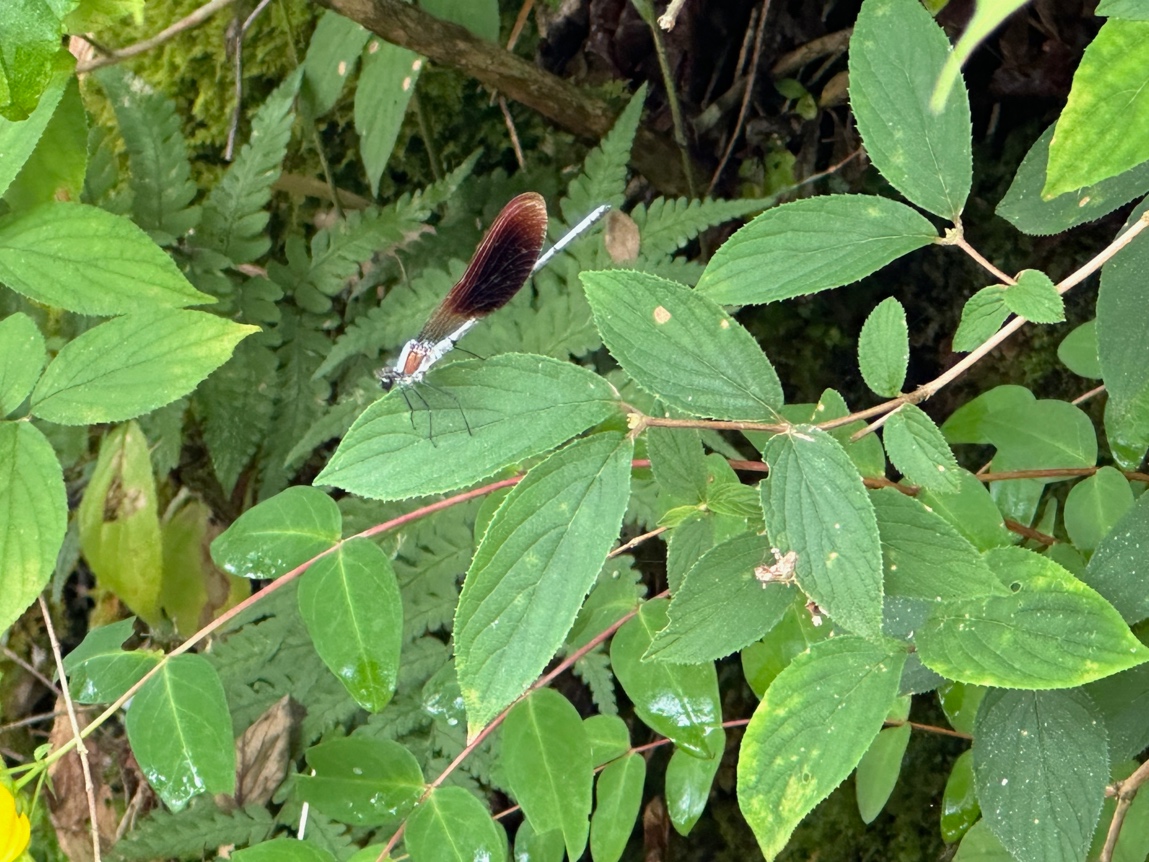
**

**Figure S4** A dark-winged territorial form of *Mnais pruinosa* in a population in Kyushu, western Japan. Populations in this area are believed to have been sympatric with *Mnais costalis* for longest (Futahashi 2017, see main text). Photo by TNS.

**A more complex version of the game**

The game considered in the current paper allowed males with different strategies to interact at non-uniform rates and females to actively choose one type of male over another. However, on occasion males may encounter a female without having to fight any other male (just as we have allowed males to interact with one another without a female being present). Here we present an extended version of the single-species game which incorporates this possibility. The model can be readily expanded to two interacting species. Note however, we have not sought to characterize the evolutionary dynamics implied by this more complex 3 x 2 game.

Let *r_N_* be the relative rates at which a territorial and sneaker males encounter a female without there being any other male present (we assume this is the same for both types of male). The revised payoff matrix now becomes:

| Payoff to row male | Interacting with | | |
| --- | --- | --- | --- |
|  | T | S | Female alone (*N*) |
| T | (($(V-d)/2)-C) m-C(1-m)$ | $\left\{ V \left( w q_{M}+\left( 1-w \right)y \right)-d \right\} m$ | $\left( V-d \right)$ |
| S | $V \left( 1-\left( w q_{M}+\left( 1-w \right)y \right) \right)m$ | $\left( \frac{V}{2}-C \right)m-C(1-m)$ | $V$ |

The payoffs to territorial and sneaker males per interaction are therefore:

$$f_{T}=\frac{x r_{TT}W \left( T,T \right)+\left( 1-x \right) r_{TS}W\left( T,S \right)+r_{N}W(T,N)}{x r_{TT}+\left( 1-x \right) r_{TS}+r_{N}}$$

$$f_{S}=\frac{x r_{ST}W \left( S,T \right)+\left( 1-x \right) r_{SS}W\left( S,S \right)+r_{N}W(S,N)}{x r_{ST}+\left( 1-x \right) r_{SS}+r_{N}}$$

The associated replicator dynamics are:

$$\dot{x}=x \left( 1-x \right)\left( f_{T}\left[ y, \right]-f_{S}\left[ y, \right] \right)$$

$$\dot{y}=y \left( 1-y \right)(g_{T}[x,]-g_{S}[x,])$$

with:

$$p_{TS}= x \left( 1-x \right) {m r}_{TS} /( x^{2}m r_{TT}+x\left( 1-x \right)m r_{TS}+\left( 1-x \right)^{2}m r_{SS}+r_{N})$$
